# Application of Various Molecular Modelling Methods in the Study of Estrogens and Xenoestrogens

**DOI:** 10.3390/ijms21176411

**Published:** 2020-09-03

**Authors:** Anna Helena Mazurek, Łukasz Szeleszczuk, Thomas Simonson, Dariusz Maciej Pisklak

**Affiliations:** 1 Chair and Department of Physical Pharmacy and Bioanalysis, Department of Physical Chemistry, Medical Faculty of Pharmacy, University of Warsaw, Banacha 1 str., 02-093 Warsaw Poland; annamazurek21@gmail.com (A.H.M.); dpisklak@wum.edu.pl (D.M.P.); 2 Laboratoire de Biochimie (CNRS UMR7654), Ecole Polytechnique, 91-120 Palaiseau, France; thomas.simonson@polytechnique.edu

**Keywords:** molecular modelling, estrogens, xenoestrogens, estradiol, docking, Density Functional Theory (DFT)

## Abstract

In this review, applications of various molecular modelling methods in the study of estrogens and xenoestrogens are summarized. Selected biomolecules that are the most commonly chosen as molecular modelling objects in this field are presented. In most of the reviewed works, ligand docking using solely force field methods was performed, employing various molecular targets involved in metabolism and action of estrogens. Other molecular modelling methods such as molecular dynamics and combined quantum mechanics with molecular mechanics have also been successfully used to predict the properties of estrogens and xenoestrogens. Among published works, a great number also focused on the application of different types of quantitative structure–activity relationship (QSAR) analyses to examine estrogen’s structures and activities. Although the interactions between estrogens and xenoestrogens with various proteins are the most commonly studied, other aspects such as penetration of estrogens through lipid bilayers or their ability to adsorb on different materials are also explored using theoretical calculations. Apart from molecular mechanics and statistical methods, quantum mechanics calculations are also employed in the studies of estrogens and xenoestrogens. Their applications include computation of spectroscopic properties, both vibrational and Nuclear Magnetic Resonance (NMR), and also in quantum molecular dynamics simulations and crystal structure prediction. The main aim of this review is to present the great potential and versatility of various molecular modelling methods in the studies on estrogens and xenoestrogens.

## 1. Introduction

Successful applications of molecular modelling methods can be found in almost every branch of modern physics, chemistry, and biology. This versatility and popularity results from the constantly increasing computing power of both personal computers and specialized servers as well as the availability of molecular modelling software. The number of properties that can be accurately predicted and phenomena that can be explained as well as problems that can be solved using such calculations are enormous. Therefore, in this review, recent advances in molecular modelling applications to study the chemistry and biochemistry of estrogens [1] and xenoestrogens [2] will be presented. The aim of this article is not only to present the large volume of information concerning title compounds that have been obtained in recent years using in silico methods but also to present to researchers who are not specialized in molecular modelling methods the possible applications of these compounds and, in that way, encourage them to use such calculations in their own studies.

This review is organized as follows: first the title compounds, estrogens, and xenoestrogens are briefly summarized, with particular focus on those that have already been objects of computational studies. Then, the most important biomolecules that are involved in the metabolism and action of estrogens and xenoestrogens are described. In the main part of this review, the computational methods that have been employed in the studies on estrogens and xenoestrogens are presented. Each of the methods is briefly described, without too many details, as there are many very good reviews referenced in this article focusing on the basics of particular methods. Wherever possible, the computational results were compared to the corresponding experimental ones; however, in many cases, such comparison was impossible either due to the lack of experimental results or the purely theoretical character of the published work. In the next section, a critical analysis of the reviewed methods is presented, supported by the presentation of some technical aspects of the reviewed studies. This was done to facilitate the choice of a certain method or its properties. From the authors’ perspective, the number of studies on estrogens and xenoestrogens is constantly increasing; however, only in some of them are the experiments supported by suitable theoretical studies. Therefore, our aim was to present to researchers working with estrogens and xenoestrogens selected computational tools that can be used to facilitate and improve their studies.

### 1.1. Estrogens and Xenoestrogens: Types, Main Representatives, and Their Toxicity

Estrogens are a group of natural steroid sex hormones. There are four of them: estrone (E1), estradiol (E2), estriol (E3), and estretrol (E4) [1] (see the Supplementary Materials: Figure S1). The last one is produced only during pregnancy by the fetus liver [3]. Among these four compounds, E2 plays the most important role in the human organism and, therefore, is of high importance in breast or ovarian cancer progression. With regard to their relative binding affinity (RBA) to Estrogen Receptor (ER), with the exclusion of estretrol, estrogens are ranked in the following order: estradiol > estrone > estriol. In comparison to estradiol, the activity and potency of estrone and estriol are, respectively, 10 and 100 times lower than that of E2 [4]. E1 mainly functions as estradiol’s metabolite and, at the same time, serves as its precursor (the estrone-to-estradiol transformation is reversible) [5]. On the other hand, in non-pregnant women, estriol levels in the blood are hardly detectable, whereas during pregnancy its amount distinctly grows because it is produced by the placenta as well [6]. All estrogens are used as medication in menopausal hormone therapy, although estradiol is the most applied [7,8]. In such external applications, they should be considered as xenoestrogens.

Apart from estrogens, other non-endogenous substances, called endocrine-disrupting chemicals (EDCs), can bind to the ER. According to the European Commission Regulation from 2018 [9], EDCs are substances that have adverse effects in non-target organisms, have an endocrine mode of action, and exhibit these adverse effects as a consequence of an endocrine mode of action. In the Guidance on the identification and studies regarding ECDs, published by the European Chemical Agency (ECA) and the European Food Safety Authority (EFSA) [10], the importance of in silico studies in the process of EDC research is clearly pointed out.

More detailed information on in silico examination regarding EDCs is provided in OECD (Organization for Economic Co-operation and Development) Guidance (Update v3, 2017) [11], where Quantitative Structure–Activity Relationship (QSAR) methodologies, prediction of the metabolic transformation (ADME), and CYP450 metabolism investigations are listed as Level 1 methods in the identification and study process. It is emphasized that applications of such methods can be used to identify the groups of chemicals and structural characteristics that are responsible for the observed in vivo effects and can serve as a tool to explain possible differences between in vitro and in vivo results, clarifying EDCs’ mechanism of action. In the Conceptual Framework for Testing and Assessment of EDCs [12], at investigation Level 1, not only QSAR and ADME but also ‘other in silico model predictions’ have been listed. Additively, special QSAR Guidance on the topic has been published.

A large group of EDCs that are well-known and have been investigated for decades are xenoestrogens, which are xenobiotics that cause either an estrogenic or an antiestrogenic effect [13]. Nowadays, they are widespread and originate from different sources (see the Supplementary Materials: Figure S2). In the in silico studies, the main emphasis has been put, so far, on the investigation of pharmaceuticals, phytoestrogens, bisphenol A, and phthalates.

### 1.2. Estrogen-Related Biomolecules as the Molecular Modelling Study Objects

Numerous studies involving various in silico methods, but mainly ligand docking, have been performed to look for new inhibitors of the enzymes metabolizing estradiol. These proteins are a possible target for new drugs and are depicted in Table 1. Most of the research deals with aromatase [14,15,16,17,18,19,20,21,22], as its inhibitors, like letrozole, are already in medicinal use [23]. However, those inhibitors exhibit some detrimental side effects, which is the reason why research continues on this topic. 17β-Hydroxysteroid dehydrogenase (17β-HSD) [24,25], sulfatase (STS) [26], as well as sulfotransferase (SULT) [27] have also been taken under close consideration using molecular modelling in order to contribute to anti-cancer drug development. The first of the two enzymes transforms estrone into estradiol. The second inactivates estradiol by transforming it into a sulfated form.

Even if conceptually estradiol is central to the above-mentioned studies, none of them used it as a target ligand. Nevertheless, in order to model estradiol activity metabolism in molecular studies, the most common objects are estradiol itself, its natural receptor—the estrogen receptor (ER)—and its main hydroxylating enzymes: CYP1A1 [28], CYP1A2 [29], CYP1B1 [30], and SHBG (sex hormone-binding globulin). The last one is a protein that can bond estradiol (E2); thus, it has direct impact on the amount of free E2 in plasma and, consequently, on the hormone’s bioavailability [31]. Taking into account the interest of in silico research for the enzymes listed in this paragraph, only topics associated with these systems are covered in the main part of this review.

Two subtypes of the estrogen receptor are known, ERα and ERβ, with tissue-specific expression [32]. ERα is found mainly in the mammary gland, uterus, ovary (thecal cells), male reproductive organs, prostate, liver, and adipose tissue. ERβ is present in the prostate, bladder, ovary (granulosa cells), colon, adipose tissue, and immune system [33]. Despite being encoded by different genes, both estrogen receptors show high homology, and in both of them the E domain contains the ligand-binding domain (LBD) and C domain, which is the DNA binding domain (DBD) [34]. The homology between ERα and ERβ in DBD is more than 95% [35]. In LBD the homology is about 55% [35]. In ER structures, two transactivation functions are present, called AF-1 (located in N-terminal domain) and AF-2 (located in LBD) [36]. They contain the nuclear location signals and, after proper exposure of their surface, are responsible for incorporation of the co-activators, which is a necessary step to induce activation of the intercellular signaling pathways.

Binding of 17β-estradiol and any agonist to the ER requires creating a hydrogen bond with His524 (in ERα) [37] or His475 (in ERβ) [38]. This leads to a unique agonist-bound conformation of the receptor’s LBD, characterized by a specific repositioning of the H12 helix, which is the most C-terminal helix of the LBD (molecular switch) [39]. On the contrary, selective ER modulators (SERMs) such as raloxifene or tamoxifen induce relocation of H12 into the co-activator binding cleft, which blocks AF-2 activity. Finally, pure antagonists completely destabilize H12 [40].

All the above actions concerning estrogens’ binding to ER are genomic actions. This means that they require translocation of the estrogen–ER complex to the nucleus and interaction with chromatin at specific sequences, known as estrogen response elements [41]. There are other estrogen signaling paths that are non-genomic and involve indirect regulation of gene expression [42]. They include activation of various protein-kinase cascades after binding of an estrogen molecule to a membrane receptor, usually GPER1 (G protein-coupled ER1) [43]. It has been proven that estrogen binding to GPER1 shows similarity to estrogen–ER binding; however, estrogen’s affinity for GPER1 is significantly lower than for ER [43]. Nevertheless, all other steroid hormones are characterized by even lower affinities towards GPER1.

Experimental analysis of the GPER1 structure has been limited, as the protein is rather refractory to both X-ray crystallography and NMR due to its relatively high lipophilicity [44]. This explains the importance of computational homology modelling [45]. Homology models are later used to simulate binding with estrogens. There are only a few studies dealing directly with this subject [44,46]. Application of homology modelling, molecular docking, and molecular dynamics provides insight into the process of estrogen binding and helps to explain the induced non-genomic effects. As non-genomic estrogen actions through GPER1 and kinase cascades alter the cell membrane shape, further research on cell membranes and estrogens is also performed (see Section 2.1.3).

## 2. Application of Molecular Modelling Methods in the Study of Estrogens and Xenoestrogens

Molecular modelling could be of a great help to experimentalists. Ligand docking directs research to the most probable hit molecules; QSAR, an officially accepted OECD method, predicts toxicity and often can facilitate research; and MD allows one to observe the time-dependent mechanisms (e.g., in membranes) and, therefore, helps to explain the experimentally observed phenomena. What is more, two types of theories on which calculations are based deliver two levels of accuracy. These are QM and MM. The former is more accurate, but as a consequence, calculations are more time-consuming. The latter, on the contrary, allows one to obtain a general view on the topic in a shorter time. The diversity of methods highly contributes to more successful experimental studies as it saves time and points out these research approaches that have a high probability of success.

### 2.1. Application to Estrogens

#### 2.1.1. Ligand Docking Using Force Field Methods

- Principles of docking and re-docking

Protein–ligand docking is a technique used to predict the orientation and active conformation of a molecule in an active center. Based on this prediction, the binding energy between protein and ligand is calculated [47]. Ligand docking can identify the chemical bonds crucial for activity and the specific atoms/residues that are responsible for them. Target proteins are usually acquired from the RCSB Protein Data Bank (PDB) [48] (Table 2). Presence of a ligand in the crystallographic receptor structure simplifies and speeds up researchers’ work, as a proper region for docking is already plainly indicated.

Accessibility of the X-ray protein structures with docked ligands gives also possibility to prove the quality of the chosen simulating docking method and parameters [49]. This is performed via extracting the ligand from the available in the PDB structure and re-docking it to this crystallographic measurements-based protein. The results are obtained in a form of the root mean square deviation (RMSD) of atom positions [49]. It is calculated as differences between the original experimental atom positions in the crystallized structure and the theoretically obtained molecular docking results [50]. It is acknowledged that the used modelling tools are able to identify the correct ligand pose, are repeatable and reliable when RMSD is smaller than 2Å [51].

Molecular modelling performed on estrogen-related receptors is not an exception. As the studies are performed mostly to search for new agonists or antagonists, the ligands re-docked into the ERs or SHBG are mainly E2 [52,53,54,55,56,57] and 4-hydroxytamoxifen [52,58,59]. RMSD value in most of the studies varies from 0.26 to 1.4Å, which proves the correctness of the docking methods in finding the proper orientation of the ligand in the active site.

- Enzymes and receptors used as targets in estrogen-related docking studies

A great many proteins have been used in molecular docking studies of estradiol. They include 17β-hydroxysteroid dehydrogenase [60], progesterone receptor [61], protein disulfide isomerase [62], SHBG [63], CYP1B1 [64], steroid sulfatase [65], and even the voltage- and Ca^2+^-activated K^+^ channel β1 subunit [66]. In all studies, an emphasis is put on the importance of OH-hydrogen binding [67,68]. The relevance of this emphasis is confirmed by experimental results. One example is a study where the estrogen analog with the highest affinity, measured in a fluorescence polarization displacement assay, appears to have the second highest predicted affinity [67].

Docking serves either to investigate the binding of estradiol to the ER or to dock other molecules, potentially new drugs, such as potent and highly selective estriol analogues [69]. In the second case, information from previous studies on estradiol behavior in an active site serves as reference data [70,71,72,73]. This is a starting point for the commonly applied research sequence: investigation of E2 binding mode, comparison with the calculation results for potential drugs, and confirmation of the hypothesis by analytical techniques. Such a three-step process has been performed, for example, for estrogen-dependent MCF-7 cancer cells [70]. Eighteen compounds with antiproliferative activity have been docked into the cavity where E2 normally binds. It was predicted that, compared to E2, an additional aromatic ring is involved in the binding mechanism. The prediction was confirmed by site-directed mutagenesis.

In some cases, allosteric modulators have been studied. This means that estradiol must be present in the binding site during the docking process [74,75]. To test the prediction of allosteric activity, in vivo experiments are often performed. For example, in one study [74] it was shown that one particular compound can properly fit into the region of the binding pocket, along with E2. Afterwards, this ligand was investigated in vivo and was demonstrated, indeed, to be a new ER-beta-selective, negative allosteric modulator of E2 binding.

Including an estradiol molecule in the docking studies helps to properly score the resulting data [76] and rank the tested molecules according to their binding affinity [77]. For large ligand sets, high-throughput screening with a pure agonist (estradiol), an antagonist (tamoxifen), and decoys (known non-binders) can be performed. This can aid in setting a laboratory’s experimental testing priority, reducing the cost and time of its research, and boosting its effectivity [77]. This explains why in vivo and in vitro investigations are often combined with in silico ones [78,79]. Altogether, it enables the discovery of new possible drug molecules that could influence estradiol’s signaling pathways [80].

- Docking studies of plant-derived potential xenoestrogens

Docking plant-derived substances into ER and comparing their binding energies and interactions (above all hydrogen bonding) with those of estradiol is a common practice. Such studies deliver information on conformational flexibility [81], the ability of the investigated molecules to selectively modulate ERα/β ability [82,83], their possible reductive influence on breast cancer risk [84], or their applicability to reduce menopausal symptoms [85]. Most importantly, data derived in this way very often show good consistency with experiments [86]. For the obtained data, a correlation with estradiol, but sometimes also with tamoxifen [75] (ER antagonist) or genistein [87,88], is found. This last substance serves as an important reference, as genistein has a higher affinity for ERβ than 17β-estradiol.

Even if docking into ER is most widely used, modulation of CYP450 activity by plant-derived substances with regard to estrogenic effects has also been examined [89]. Indeed, the same signaling pathways are regulated by CYP450–estradiol interactions, and there are plenty of data available on this topic. Moreover, other calculation methods are also applied, including ADMET and molecular dynamics (MD) [90,91] (for MD description and examples, see Section 2.1.3). Nowadays, in silico methods are a standard tool in plant xenoestrogens studies. Thus, molecular modelling is often performed in parallel to either in vitro [92] or in vivo toxicity studies [93], and in most cases an agreement between data obtained from both sources is found. This was the case in the docking-based binding affinities of compounds derived from *C. elegans* and their measured reproductive toxicity [93]. Wide examination of many substances of a natural origin is possible thanks to the thorough structural knowledge on the estradiol molecule bound to the ER (Table 2).

#### 2.1.2. Quantitative Structure–Activity Relationship (QSAR)

QSAR methods use mathematical models to correlate structural characteristics with the biological activity of a set of compounds that have closely related structures [94]. Empirical and theoretical molecular descriptors are used. Conceptually, three main types of QSARs are known: ones based on fragment analysis of a system (here, a pharmacophore [95] concept is used), ones that consider the given system as a whole (descriptors are computed from scalar quantities), and 3D-QSAR. In this last type, descriptors are obtained by application of a force field (3D approach). To achieve a 3D target structure, either software-based alignment or manual superimposition on the crystallographic data must be performed [96]. This is a necessary step, as different ligand-binding modes and bioactive conformations are possible. Examples of 3D-QSAR are Comparative Molecular Field Analysis (CoMFA) [97] and Comparative Molecular Similarity Indices Analysis (CoMSIA) [98]. From the created QSAR models, predictions on the bio-activity and toxicity of other molecules are made.

A smaller group of structure–activity studies are ones that are non-quantitative, namely SAR ones (structure–activity relationship). They include virtual screening with a pharmacophore and a large set of molecules. Afterwards, in vitro evaluation of the data is performed. This methodology has been used to determine potential 17β-HSD inhibitors [99,100] that could be applied in the treatment of osteoporosis provoked by estradiol deficiency.

Among all molecular modelling approaches, QSAR is one of the most commonly used to examine estradiol’s structure and activity. Often, data on estradiol serve only as a reference for information gathered on new possible drugs [14,101] such as raloxifene derivatives [102] or estradiol metabolites. Through comparison with experiments, it has been shown that QSAR models have a high sensitivity and specificity in providing relative binding affinities (RBAs), where estradiol’s RBA equals 100% [103]. Among the most important descriptors are ones calculated with quantum mechanics at the DFT level [104,105].

While QSAR is mostly applied to ER-binding, other proteins like CYP1A2 [106] and CYP1B1 [107] have also been targeted. One study used the CoMFA approach to model estriol’s influence on CYP1A1 [106]. It focused on the inhibition of estradiol to mutagenic 4-OH estradiol transformation. Out of 90 steroid candidates, thioestrone was selected and shown to have the desired inhibitory ability. Its mechanism of action was revealed by molecular modelling. It is desired because thioestrone’s -SH group is closer to the iron atom in the CYP1B1 heme than the -OH group in natural ligands of the enzyme, namely estradiol and estrone.

OECD Guidance identifies QSAR as an important element in toxicity evaluations. As a result, QSAR plays an important role in substance risk assessment [108]. It has been applied in studies dealing with mutations in enzymes that metabolize estradiol [109] and to study estradiol oxidation and emerging contaminants [110]. In the latter study, the most accurate DFT descriptors were used. This enabled a better understanding of the degradation mechanisms.

#### 2.1.3. Advanced Docking Using Combined Quantum Mechanics/Molecular Mechanics (QM/MM) or Molecular Dynamics (MD) Methods

Molecular modelling with QM is much more accurate than MM methods. However, the direct use QM approaches in drug design is limited due to the cost and size of protein structures. In recent years, QM/MM has been gaining attention, as it allows one to consider a whole protein–ligand complex and not only a binding site [111,112]. Due to computational limitations, MM calculations on the outer part of a receptor deliver only approximate data. Nevertheless, QM/MM provides more knowledge on the protein’s influence than is obtained when only the LBD region is considered.

More commonly applied methodology is MD. It simulates time-dependent processes and provides data that are otherwise unavailable [52,54]. For example, in case of ERs or SHBG ligands MD can point out whether the examined substance is receptor’s agonist or antagonist. This assumption is based on the RMSF (root mean square fluctuation) value which is extracted from the MD trajectories. RMSF represents the flexibility of the amino acid residues [50]. Both RMSF and RMSD depend on the interactions between the protein and the ligand and are the result of the ligand movements in the active site trying to achieve the appropriate position [63]. If the same ligand undergoes the MD process in both ERα and ERβ and its RMSF and RMSD values significantly differ for these two receptors, it indicates that the investigated molecule probably occupies more favorably one of the investigated receptors. Such data leads to the hypothesis of the ligand’s selectivity.

Moreover, in terms of the ERs, RMSF and RMSD values suggest whether the analyzed molecule is the receptor’s agonist or antagonist [50,52,53,54,59]. As already mentioned in Section 1.2, the positioning of the H12 helix is differently influenced by agonists and antagonists. If the estrogenicity of the compound is known, comparison of RMSF values with the known molecule’s estrogenic effect, can serve as an evaluation of the applied docking parameters.

Nevertheless, MD requires a lot of computation time, which increases with the system’s size and simulation length. In return, it describes the dynamics of the system as well as entropic effects associated with the protein–ligand interaction.

MD can also provide quantitative estimates of relative binding affinities through techniques known as “free energy calculations”. The most rigorous is the “free energy perturbation,” or FEP family of methods [113,114,115,116,117]. To compare two ligands, say A and B, one introduces a model where both ligands are present, each with a partial occupancy. This is closely analogous to a crystallographic refinement where a particular group (ligand or side chain, say) has two possible conformations. Each ligand is assigned a weight between 0 and 1, say w_A_ and w_B_ = 1 − w_A_. These multiply terms in the energy function involving either ligand. By varying the weights gradually, one can effectively remove one ligand and introduce the other. Thus, when w_B_ = 0, ligand A is fully weighted while interactions of ligand B with its surroundings have a zero weight: ligand B is “invisible” to its environment. Usually, interactions within the ligand are not weighted. As w_B_ changes from zero to one, B is introduced and A is removed. Intermediate weight values correspond to an “alchemical” mixture, where both ligands are partially present. MD simulations (or Monte Carlo simulations) are performed for a few w_A_ values, typically around 10. This series of simulations mimics a gradual, reversible replacement of A by B. Energy statistics are collected from all simulations. From these, a free energy difference between A and B can be obtained. The same process is carried out for the unbound ligands, solvated by a box of water. Subtracting the bound and unbound free energy changes yields the binding free energy difference. The method requires force field parameters for each ligand but has no other adjustable parameters. The tradeoff is that not one, but several MD simulations are required, and these should be sufficiently long. Indeed, FEP accuracy is limited by the MM force field, but also the amount of conformational sampling that is carried out. The method can also be used to compute the binding free energy changes due to point mutations of the protein. In this case, a particular residue is modeled with two side chains, each having a partial occupancy. Nowadays, FEP can be applied to one ligand or mutation per day on a medium-sized computer cluster.

A simplified version of FEP is to use only two simulations per ligand: one bound to the protein and one in solution. From these, binding free energies can also be obtained, if one is willing to extrapolate from a w_A_ value of unity to a value of zero. To counter the use of such a large extrapolation, one introduces empirical weighting factors that multiply the interactions between the ligand and its surroundings (protein or solution). Usually one is applied to the electrostatic interactions and one to the Lennard–Jones interactions. The extrapolation and use of interaction energies have led to the name Linear Interaction Energy, or LIE method [118,119,120]. For a thorough review of its theoretical basis, see [121]. The tradeoff for its speed is that experimental data are needed to adjust the values of the empirical weights, which are not very transferable between different proteins and classes of ligands. Once the weights are optimized for the molecules of interest, predictions can be made.

In many studies, estradiol has been simulated in a complex with ER. The QM/MM approach seems to be the most accurate. It uses a QM description of the ligand and its binding pocket, but thanks to the MM description of more distant protein regions, it preserves information on the whole enzyme’s impact [122]. Very recently, QM/MM elucidated the important role of estradiol’s D-ring in the active site of ERα [123]. What is more, it helped to understand the influence of each enzyme segment on the ERα-agonist (estradiol, diethylsilbestrol) binding [124]. Most importantly, the binding energies of E2 and DES correlated well with experimental agonist binding affinities for the ER.

To analyze the changes in the investigated complexes upon DNA binding, not only MM, as with estradiol and DNA [125], but also MD has been applied [126]. The latter study identified specific bases within the aptamer (short-stranded DNA/RNA, binds only specific molecules [127]) and demonstrated the importance of water-mediated hydrogen bonds in the aptamer–estradiol complex.

MD is also often used to describe the effects of enzyme mutations on ligand binding. This methodology has been applied in estradiol studies, to compare wildtype and mutated CYP1B1 [128], which is mostly responsible for the 2-OH-hydroxylation of estradiol. MD serves also as a tool to explain the results of molecular docking. For example, it has been stated that the interactions between human α-fetoprotein and agonists (estradiol, estrone, diethylsilbestrol) were caused by van der Waals forces, whereas binding of antagonists (tamoxifen and its analogues) was equally based on hydrophobic and electrostatic interactions [129]. Moreover, information from MD simulations can be used to construct a pharmacophore in order to screen protein databases for a desired type of ligand. This methodology was used to search for substrates and inhibitors of the estrone-SULT [130]. Nine selected molecules were consistent with the ones indicated by the experiment.

Another nuclear receptor, the farnesoid X receptor (FXR), was used as a test case for free energy calculations in 2018 [116,117,120]. FXR is involved in regulating bile acid, lipid, and glucose homeostasis [131], and it has been linked to hepatocarcinogenesis [132]. Its hormone binding site is hydrophobic with few conserved interaction motifs and strong induced fit effects. With FEP, mean errors were about 1.5 kcal/mol for relative binding free energies of around 30 ligands, and the largest errors were about 2.5 kcal/mol. In one of the studies [117], ligands were first docked to the receptor, then compared using FEP. LIE gave similar errors for the same protein and 47 ligands [120], at a lower computational cost, but required optimization of the two adjustable LIE parameters using a subset of the ligands.

An earlier LIE study [119] considered the ER binding of estradiol and a series of xenoestrogens. A training set of 19 ligands was used to optimize the LIE parameters. A mean unsigned error of 0.6 kcal/mol was then obtained for a test set of 13 ligands. Several binding poses (3–4) were considered for each ligand; this was not too expensive because only short MD simulations were run.

#### 2.1.4. Other MD-Based Studies of Estrogens

- Membranes

As ERs are located in either the cytoplasm or the lipid bilayer (mER, membrane ER) [27,133], and estradiol itself is a steroid hormone, closer insight into the ligand’s interaction with the cell membrane is an obvious research target. Since transfer through this cellular barrier is not a stable state, but a dynamic process, MD calculations could be seen as a method of choice. Recent studies revealed [134] that, regarding estradiol’s long axis and the lipid acyl chains, E2 adopts a perpendicular position in the membrane. By having four rings located near the membrane interface, participation of the hormone’s 3-OH and 17β-OH groups in hydrogen bonds and electrostatic interactions with the lipids are possible.

Combining MD and QM enables further research into E2 membrane crossing. It provides information not only about the E2 orientation in the membrane (MD) but also about the strength of the electrostatic potential mapped on the electron density surface (QM) [135]. These data, derived from a HDL disc model, enable deeper insight into the mechanism of E2 incorporation into lipid membranes and is an important step forward to develop tissue-specific discs encircled by a membrane, which would serve as transporters for E2 or its derivatives.

Similar methodology (QM, MD) was used to study the removal of hormonal pollutants from water. It has been shown that by using high levels of salinity, which increases the strength of hydrogen bonding and hydrophobic interactions, one can perform a membrane-based sorption of 17α-ethinyl estradiol on the polyethersulfone membrane [136].

- Nanotubes

Nowadays, estradiol is a relatively common water pollutant, and numerous studies have been performed to find a reliable tool for its removal. One possibility is to use nanotubes. For that purpose, within the molecular modelling approach, mostly single-walled carbon nanotubes (SWNTs) [137] are used. Free energies of adsorption have been calculated with a QM approach. In some cases, MD was also implemented [138]. The target ligands for these studies were 17β-estradiol and its medically useful derivative 17α-ethinyl-estradiol [139,140,141]. These simulations revealed the adsorption energy, a preferential sorption among different nanotubes and estradiol derivatives, and provided a molecular explanation for the observed results [142].

#### 2.1.5. Density Functional Theory (DFT) Calculations in the Study of Estrogens

DFT is a QM approach that determines the ground-state properties of a many-body system by applying the electron density concept. The underlying concept is the Hohenberg–Kohn theorem [143], later developed into the Kohn–Sham theory (KS-DFT) [144]. Firstly, the energy of the system for a non-degenerate stationary state is uniquely determined by its electron density, which depends on three spatial coordinates. For this reason, the energy is expressed as a functional of the (scalar) electron density function. Secondly, according to the H-K theorems, the minimum energy occurs for a unique, precise electron density in the ground state. KS-DFT includes the Coulomb interactions between electrons and considers the energy of the exchange and correlation interactions. For a long time, the dispersion energy (the energy of the long-ranged electron correlation) [145] represented a difficult problem, as it is a time-dependent phenomenon, and it was not included in KS-DFT. However, nowadays, dispersion corrections are available and can be included in DFT functions [146]. Therefore, application of DFT leads to the highest obtainable accuracy in calculations. The only existing drawbacks are the risk of underestimating the energy [147] and the time needed to acquire the results. DFT-based calculations are especially widely used in solid-state studies.

Although application of DFT is mostly concentrated on the investigation of single molecules (see the paragraphs below), it is also used to determine total binding energies between a ligand and protein. This is the case for systems composed of ER, SERMs, and two widely used ER antagonists: 4-hydroxy tamoxifen (4OH-T) and raloxifene (RAL) [148]. The results show that the 4OH-T-SERMs set binds more strongly to the ER than the RAL-SERMs set. This is fully in agreement with the experimental data and, once more, as many other studies, confirms the high accuracy of the DFT calculations.

- Crystal structure prediction

DFT is the theoretical basis for periodic calculations performed on solids, often pharmaceuticals, in order to find and depict new polymorphic forms of drugs or potentially bioactive molecules [149]. It can also be a part of the Crystal Structure Prediction (CSP) approach. Such methodology has been recently used to study crystal structures of 17β-estradiol. As a result, an estradiol hemihydrate has been computationally determined [150]. DFT calculations are also often necessary to refine a crystal structure obtained from powder X-ray diffraction (PXRD) experiments. These calculations are mostly consistent with experimental data, as in the case of estradiol ethinyl cocrystals [151].

DFT-based methodology has also been applied to examine the dissolution process in a study of estradiol cocrystals [152] and to calculate the free energy of solvation in the estradiol–ER complex [153].

- NMR and vibrational properties calculations

**Figure 1:**
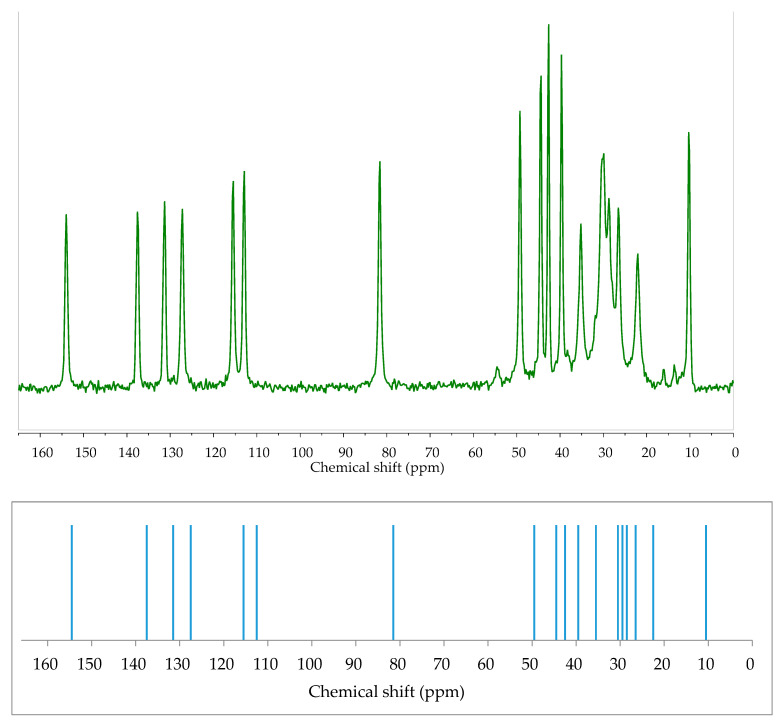
Experimental (top, green) Cross Polarization Magic Angle Spinning (CP MAS) and calculated (bottom, blue) GIPAW ^13^C solid-state NMR spectra of E2. Very good agreement between calculated and experimental values proves the usefulness of DFT calculations in solid-state analysis of estrogens. More details in [158]. Source: Author’s archive.

One of the most common DFT applications is to predict NMR and vibrational data. For NMR properties, GIAO [154] and GIPAW [155] methods are implemented. Estradiol being relatively complex (many atoms, presence of both aromatic and non-aromatic rings) could cause computational problems. For this reason, it has been part of a many-ligand study to prove the applicability of the GIAO method as well as its ability to calculate J_HH_, J_HC_, and J_CC_ NMR coupling constants [156,157]. Generally, the results obtained were satisfying. However, nowadays it is more common to study the solid state with the second method. GIPAW NMR calculations were also done recently for a new polymorph of 17β-estradiol [158]. The calculations helped to improve the assignment accuracy of chemical shifts obtained from the experiment and, therefore, to elucidate the structure of a new anhydrous estradiol form Figure 1.

Another study showed the applicability of DFT-NMR to explain the nature of a more complex system: the transversal distribution of 17β-estradiol in lipid membranes [159]. NOESY 2D NMR spectra contained cross-peaks between the hormone and lipids. Here, too, DFT calculations helped to properly interpret the experimental data and, as a consequence, greatly aided in describing the position of the estradiol aromatic ring in the membrane. An implication of such study is to increase our understanding of estradiol’s transfer through a lipid bilayer [159,160]. A second area where DFT methodology is widely applied is in calculating vibrational properties. The direct usefulness of the method is manifested through its contribution to accurate assignment of the vibrational modes in IR or Raman studies. This has been implemented, for example, in investigations of estrogens [160] and estradiol-17 valerate [161]. In most reported cases, computationally generated spectra were in very good agreement with the experimental data.

**Figure 2:**
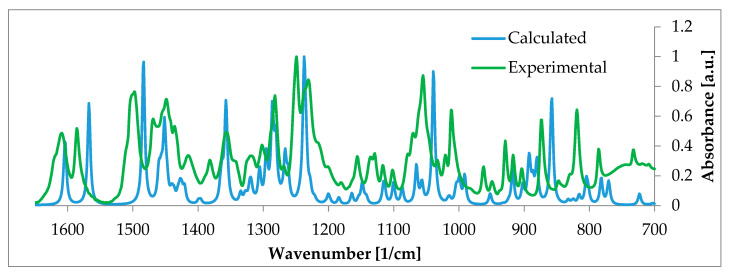
Experimental (green) and calculated (blue) IR spectra of β-estradiol hemihydrate, selected range 700–1650 cm^−1^. Such calculations enable proper band assignments and thus facilitate the spectrum analysis. More information can be found in [158]. Source: Author’s archive.

From a wider perspective, DFT calculations enable one to properly describe the examined subject. Additionally, in case of estrogens and estrogen derivatives DFT-derived spectra (both vibrational [162,163] and NMR [158]) stay in a good agreement with the experimental data and are often the only way to properly assign bands (Figure 2). In order to obtain theoretical spectra which ideally meet the experimental ones, scaling factors must be implemented [164]. Thanks to such combination of the theoretical and experimental approach, the first full interpretation of estriol IR spectrum could have been published [164]. The analogical situation has been reported for E1, E2, E3, and ethynylestradiol Raman spectra [163]. Simulation was necessary to identify unique marker peaks in the finger-print region what was useful to differentiate between very similar estrogen structures.

Another example shows that the vibrational frequency calculations of estradiol alone and in monohydrated form [165] has given insight into hydrogen bond formation by estradiol’s D-ring. This has been used to discuss the relationship between the stability of hydrated clusters and the estradiol conformation. In another study [166], DFT-based IR and Raman frequencies were used to investigate estradiol and tamoxifen structures. This helped to understand the intermolecular interactions made by these two molecules and to interpret opposite estrogenic effects. However, it should be mentioned that in the studies of estrogens, Raman spectroscopy is used less frequently than IR. This is due to the inherently weaker signals and common presence of fluorescence interference from the contaminants [163]. Apart from IR and Raman, low-frequency vibrations could also be calculated with the help of DFT. A good example is the assignment of vibrational modes in terahertz spectra for testosterone, estradiol, and estrone [167].

- Removal of estrogenic pollutants

With regard to estrogen removal from the environment, QM calculations are performed not only on nanotubes but also on other sorbents (e.g., lignocellulosic material) [168]. The adsorption energy shows that this adsorbent could be used to remove all three main estrogens, E1, E2, and E3, from a solution by means of liquid phase extraction. Another extended study revealed the applicability of reduced graphene oxide modified with silver nanoparticles in electrochemical detection of estriol [169]. Firstly, MD simulations at 1000 K were performed in order to obtain 100 conformers of estriol. Later, these structures were used in a semi-empirical Hartree–Fock geometry that was pre-optimized with solvent simulated via the conductor-like Screening Model COSMO [170]. Afterwards, the most stable conformers were fully optimized by the DFT-based software. In this step, the solvent was included via application of the Polarizable Continuum Model [171]. Then, on the structure with the lowest energy, MD at 300 K was performed. Electric properties of the newly obtained conformers were determined with DFT calculations. This study is a good example of the wide range of in silico methods that can be applied to molecularly model the investigated subject (i.e., to develop a method to detect estriol in tap water and urine samples).

### 2.2. Application of Various Molecular Modelling Methods in the Study of Xenoestrogens

#### 2.2.1. Various Molecular Modelling Methods Applied in Xenoestrogen Studies

The same methods as for estradiol, above, are also used to investigate xenoestrogens. They include QM/MM [172] and QSAR approaches [173,174,175] and confirm the importance of the hydrogen bond between xenoestrogen molecules and the His524 residue in the active site. A variety of xenoestrogens, such as bisphenol A and C, butylparaben, 4-octylphenol, DDE, phthalate, zaeranalol, estradiol, 4-OH-tamoxifen [176], and several proteins (ER [177], SHBG [178]), have been studied. These studies either looked for structural similarities between different ligands or focused on one specific molecule, such as zearalenone [69]. The latter study suggested a lack of agonistic activity against the ER due to the lack of any stable, functionally active conformation of the tested molecule in the LBD. On the other hand, according to QSAR analyses performed on zearalenone analogues [179] and metabolites [180], these zearalenone-related compounds show some estrogenicity due to the presence of a keto/hydroxyl group, a trans double bond in the macrolide ring, and two hydroxyl groups in the aromatic ring, which participate in binding to ER.

As endocrine-disrupting chemicals are present in the environment, research into their removal is constantly being performed. For example, to study dioxin adsorption on graphene, DFT [181,182] and MD [183] calculations have been undertaken.

#### 2.2.2. Bisphenol A

- Bisphenol A (BPA)–ER complex studies

Bisphenol analogues are among the most studied xenoestrogens. Molecular modelling helps show how these closely related structures adopt agonist/antagonist orientations in the estradiol binding pocket [184] and delineate the binding modes of each bisphenol molecule [185]. A deeper study, concentrating not only on the pure ligand–receptor binding but also including the allosteric effects and application of MD calculations, showed that BPA causes changes in a full-size receptor, and its effect is not limited to the separate domains [186].

A separate set of studies investigated BPAs with halogen substituents on the phenolic rings. All studies showed that a hydrogen bond with His524 for an agonist and with Thr347 for an antagonist was created, exactly as in the case of estradiol and 4-OH-tamoxifen, respectively [187,188]. What is more, just as for estradiol, the stability of helix H12 is crucial in halogenated BPAs–ER complexes [188,189]. The in silico results have been confirmed by experimentally measured affinities.

Although BPA mimics estradiol’s action in the LBD, the QM/MM study revealed that, in comparison to other tested EDCs, it exhibited lower estrogenic activity, probably due to the lack of interaction with His524 [190]. In turn, application of MD helped to elucidate mechanisms driving BPA–ER binding. According to that study, direct hydrogen bonds and hydrophobic interactions are responsible for the binding [173]. Like the previously mentioned experiment, this one confirmed that the ER binding affinity is slightly lower for BPA than for estradiol. MD not only helps to elucidate bound conformations and binding energies between LBD and BPA in the ER [191], but it also gives insight into the influence of that binding on the whole protein, including the DBD. One of the studies [185] showed that the allosteric effects in the LBD due to BPA binding could cause relaxation of the DBD and, therefore, alter ER’s function. Other researchers reported the influence of bisphenol compounds on the protein’s allosteric modulation, altering the Helix12 stability and reducing the recruitment potency of co-activators [187]. This knowledge can be useful in the process of estimating the toxicity of compounds.

- Risk assessment and removal attempts

In 2012, a protocol for in silico risk assessment of BPA on the ER was proposed [189]. Later, many studies dealing with BPA removal from water were performed [192,193]. Applied molecular modelling techniques include DFT and MD. Since BPA results from the depolymerization of, for example, polycarbonates, both BPA and the initial polymer have been studied under periodic boundary conditions (pbc) [194]. The same DFT-pbc approach has been used to model the photocatalytic degradation of BPA caused by cobalt-doped BiOCl nanosheets [195] and by the effect of humidity combined with UV irradiation [196]. Another example is the evaluation of BPA’s binding to microextraction coatings [197]. In this case, dispersion-corrected DFT was applied.

#### 2.2.3. Phthalates

The second group of xenoestrogens most widely examined by molecular modelling are the phthalates [198,199]. In silico investigations showed that estradiol has a lower ER binding affinity than phthalates. The highest RBA is exhibited by monophthalates [200]. In the case of SHBG binding, score values suggest that short-chain phthalates are more potent than long-chain ones [201]. This agrees with known experimental data.

To look for an effective method of phthalate removal from water, as with estradiol, DFT and MD calculations on SWNT–pollutant complexes have been performed [202]. The adsorption energy has been calculated, and the adsorbent’s chemical groups responsible for the binding have been determined. MD has also been used to examine polymer–solvent interactions while looking for a new, more eco-friendly substitute for plasticizers [203,204].

#### 2.2.4. Technical Aspects of Calculations Performed on (xeno)Estrogens

The computational method most commonly applied in the analysis of (xeno)estrogens is molecular docking. Available publications show that, for this purpose, the most common software packages are Maestro Schrödinger and AutoDockTools. The former is also widely applied in Virtual Screening Workflow (Table 3, N° 9–11), which includes high-throughput virtual screening (HTVS) [205] and molecular docking with either standard or extra precision (SP, XP). In both cases, the OPLS 2005 force field is used. The mentioned (N° 9) consensus score is an effective score that enables ranking of the investigated ligands. It is a combination of different scores, like DockingScore (GlideScore + state penalties for protonation) [206], MM/GBSA Score (binding free energy calculations based on the MD trajectories) [207], and QSAR Score.

Virtual Screening Workflow makes it possible to screen large sets of ligands. It helps to differentiate between ‘actives’ (compounds active against the target protein), ‘decoys’ (compounds of known non-activity against the target protein), and ‘inhibitors/activators’ (potential bio-active substances). As a consequence, the Virtual Screening Workflow approach guides future ligand synthesis and helps in setting a priority for in vitro testing.

Often, a computational step following molecular docking is MD. Here, the most applied codes are GROMACS and AMBER using CHARMM and AMBER force fields, respectively. In most cases, the TIP3 (transferable intermolecular potential with three points) solvent model is applied [242].

Computational methods applied in (xeno)estrogens studies that deal not with a solvent environment but with a solid state are Crystal Structure Prediction (CSP) (N° 26, 27) [235,236,237] and DFT-based calculation of spectroscopic (IR, Raman, NMR) properties (N° 28–34). For the latter, the most commonly applied codes are CASTEP and Gaussian with GGA PBE [239] and B3LYP [241] functionals, respectively. These two functionals seem to be the most reasonable for the investigated subjects. GGA PBE establishes the non-homogeneity in the electron density and leads to more precise results, which in turn is of high importance in NMR spectra calculations. B3LYP is a hybrid functional based on combining DFT and Hartree–Fock theories and finds its application in spectroscopic spectra generation.

On the contrary, CSP [235,236,237] is a multi-step and much more complicated methodology, as it implements both MM and QM and sometimes even MD. Firstly, MM calculations are performed to generate and rank possible compound conformations. Afterwards, the selected conformers are subjected either to ab initio calculations on a molecule or to DFT-D (dispersion corrected) [146] calculations performed on the whole crystal structure. This enables one to observe conformational polymorphisms in the first case and packing polymorphisms in the second. The lattice energies obtained could be adjusted if kinetic factors (like temperature) are included. For that purpose, time- and computational power-consuming MD must be applied.

In contrast to molecular docking or MD simulations, one of the most important calculation methodologies for (xeno)estrogens, QSAR, is independent of the protein and based solely on the ligand structure. This explains why different codes must be applied for QSAR. Their application to (xeno)estrogens has already been described in detail, and the available codes have been compared in [14].

The above-mentioned codes and parameters cover the most common calculations. However, it is impossible to point out the best ones due to the insufficient number of studies. We can only observe that, out of the gathered data, some standard methodologies emerge.

However, a comparison of the applied methodologies in terms of their usage as well as their advantages and drawbacks is possible. The most important aspects have been gathered in Table 4.

To predict the binding affinities or the interactions between the (xeno)estrogens and biomacromolecules, either simple molecular docking or more sophisticated methods such as QM/MM, MD/MM, or FEP can be used. Notably, the more sophisticated methods require not only more specialized software but more computational time and power. Since, to the best of our knowledge, no study has been reported comparing the accuracy of various ligand docking methods applied to the particular group of (xeno)estrogens, no specific indications can be provided.

When focusing on the structural and physicochemical properties of estrogens and xenoestrogens, DFT-based methods have proven their high accuracy and reasonable calculation time. Therefore, such computations can be performed to obtain structural, spectroscopic (IR, Raman, NMR), and thermodynamic data of estrogens, xenoestrogens, their complexes, and solid-state forms such as solvates, salts, and co-crystals. Standard DFT functions (B3LYP for isolated compounds and PBE for periodic structures) have been found to be accurate in multiple studies.

## 3. Conclusions

In this review, it was clearly shown that molecular modelling methods are valuable tools in studies on estrogens and xenoestrogens. Their relatively low cost, requiring only certain specialized software licenses and computing servers, their increased personal and environmental safety, and their reasonable accuracy make molecular modelling methods unique and modern tools for these studies. In this article, the most common biomolecules studied using molecular modelling were presented, together with appropriate references to the published results. This group of molecules is composed mostly of enzymes participating in the metabolism of estrogens, along with estrogen receptors and even specific nucleic acid domains. While most studies focused on predicting the affinities of small molecules (ligands) to the chosen receptors, the computational research is not limited to this aspect. Another important role for modelling is to explain the conformational changes resulting from binding. Such in silico studies would not be possible without the very large number of already deposited, high-quality crystal structures of estrogen-related proteins that can be easily accessed and used in molecular modelling studies. An overview of those structures was presented in this review. The oldest studies in which molecular modelling was used to study the biochemistry of estrogens focused on molecular docking with molecular mechanics. More recent studies have used more sophisticated methods such as molecular dynamics or combinations of quantum mechanics and molecular mechanics. Further, in this review, it was shown that computational studies concern not only interactions between estrogens and biomacromolecules, but they also can be used to describe phenomena such as migration of estrogens through lipid bilayers or their adsorption on various materials. This can help predict the most efficient way to remove them from the environment when treated as pollutants. Further, it has been shown by multiple examples that quantum molecular modelling methods, such as those based on density functional theory, can be successfully used in structural studies on new solid forms of estrogens such as salts, co-crystals, hydrates, and polymorphs as well as on the complexes of estrogens with other molecules (i.e., cyclodextrins). In addition, the possibility to accurately calculate vibrational and NMR properties can be very helpful to explain spectroscopic results. Finally, we presented similar molecular modelling studies on xenoestrogens such as Bisphenol A and phthalates. Therefore, taking into consideration the versatility and confirmed accuracy of molecular modelling methods, it is not surprising that they have been listed in the specific guidance for studies on EDC published by international organizations such as ECA, EFSA, and OECD.

## Figures and Tables

**Table 1:** Reagents of the main metabolic processes regarding estradiol that have been investigated with in silico methods.

Substrate	Enzyme	Product	Ref.
Testosterone	Aromatase (CYP219A1)	Estradiol	[16]
Estradiol	17β-OH-dehydrogenase (17β-HSD)	Estrone	[24,25]
Estradiol	CYP1B1	4-OH-hydroxylated estradiol	[30]
Estradiol	CYP1A1, CYP1A2	2-OH-hydroxylated estradiol	[28,29]
Estradiol	Sulfotransferase (SULT)	Inactivated estradiol (sulfated)	[27]
Inactivated (sulfated) estradiol	Sulfatase (STS)	Activated estradiol	[26]

**Table 2:** Selected proteins involved in the metabolism of estrogens present in the RCSB PDB (Protein Data Bank) [48]. All structures were obtained using X-ray diffraction.

Protein	RCSB PDB Reference Code	Resolution (Å)	Incorporated Ligands
βER	5TOA	2.5	Estradiol
βER-LBD	1QKM	1.8	Genistein
Phosphorylated βER-LBD	3OLL	1.5	Estradiol, N-peptide linking
αER-LBD	3UUC	2.1	Bisphenol C
αER-LBD mutant	4Q50	3.07	4-hydroxytamoxifen
αER-LBD mutant	2QXS	1.7	Raloxifene
αER-LBD	2R6Y	2.0	SERM
17β-HSD	1IOL	2.30	17β-estradiol
17β-HSD	6MNC	2.40	Estrone
17β-HSD	6MNE	1.86	Estrone, NADP+
17β-HSD	3DHE	2.30	Dehydroepiandrosterone (DHEA)
17β-HSD	4FJ0	2.2	3,7-dihydroxy flavone
17β-HSD	4FJ1	2.3	Genistein
SULT1E1	1AQU	1.6	Estradiol, PAP cofactor
SULT1E1	4JVM	1.994	Flame retardant, PAP cofactor
Placental E1/DHEA STS	1P49	2.6	l-octylglucoside, *N*-acetylo-d-glucosamine, Ca^2+^, PO_4_^3−^
CYP1B1	6OyV	3.101	Estradiol

**Table 3:** Selected technical computation data in terms of ER and (xeno)estrogens regarding the publications cited in this article.

N°	Code/Software Used	Force Field or DFT Functional and Basis Set	Type of Calculation	Ref. Method	Ref. in Article
1	GOLD		Molecular docking	[208]	[57,69]
2	-Ghemical 2.95 -Swiss Dock	-Tripos 5.2 -CHARMM	-Geometry optimization -Molecular docking	[209,210,211,212]	[70]
3	-Swiss model -Hex 8.0, HADDOCK	-OPLS	-**Homology of receptors** -Molecular docking	[212,213,214,215]	[87]
4	-Swiss model -SybylX	-Tripos 5.2	-**Homology of receptors** -Molecular docking	[209,214,216]	[84]
5	Maestro Schrödinger	OPLS 2005, Glide SP, XP	Molecular docking	[217,218]	[83,85,86]
6	Maestro Schrödinger	MMFF94	Geometry optimization, molecular docking	[217,218,219]	
7	-Gaussian09W -AutoDockTools	-B3LYP/6-31G(d) -AutoDockZN	-Geometry optimization -Molecular docking	[220,221,222,223]	[169,184]
8	Gaussian03	B3LYP/6-311++g**, PCM	Hydration enthalpy	[220,224]	[183]
9	Maestro Schrödinger	ZINC database OPLS 2005, Glide SP eHiTS docking module consensus score	Energy minimization **HTVS** rank	[205,217,218,225]	[75]
10	Maestro Schrödinger	OPLS 2005, Glide SP, XP	**HTVS**	[205,217,218]	[76]
11	Maestro Schrödinger	OPLS 2005, Glide SP, XP	**Segregation**: agonists/antagonists	[217,218]	[77]
12	Maestro Schrödinger	-OPLS 2005, Grid (Glide) -Desmond OPLS 2005	-Molecular docking, MD -ADMET parameters	[217,218]	[90]
13	-Maestro Schrödinger -AMBER14 -AMBER14	-OPLS 2005, Glide -FF03 (protein) GAFF (ligand) -MMPBSA, MMGBSA	-Docking -MD -Binding free energy, decomposition energy	[207,217,218,226,227,228]	[91]
14	-MOPAC2016 -Gaussian09 -Gabedit package	-PM6 in HF, COSMO model -B3LYP, PCM -Verlet algorithm	-Pre-optimization, solvent model -Optimization (DFT), solvent model -MD	[156,220,224,229,230]	[169]
15	-GOLD -GROMACS -Swiss Param Tool	-CHARMM27 -CHARMM27	-Molecular docking -MD -Ligand parametrization	[209,210,211,231]	[180]
16	-SybylX -AMBER11 -AutoDock 4.0	-Tripos 5.2 -AMBER -AutoDockZN	-Geometry optimization -MD -Molecular docking	[216,221,222,223,226,227,228]	[186]
17	-Gaussian09 -LeDock -AMBER12 -AmberTools14	-B3LYP/-cc-pVTZ -CHARMM -AMBER -MM/GBSA	-Geometry optimization -Molecular docking -MD -Binding free energy	[207,220,221,222,223,226,227,228]	[188]
18	-Gaussian09 -Molegro Virtual Docker -AMBER Tools	-B3LYP/6-311++G(d,p) -AMBER -AMBER03	-Molecular electrostatic potential -Molecular docking -MD	[220,226,227,228]	[187]
19	-Maestro Schrödinger -Gaussian09 -AMBER10	-OPLS 2005 -HF, 6–31G* -GAFF (ligand), ff03 (protein)	-Molecular docking -Geometry optimization -MD	[217,218,220,226,227,228]	[189]
20	-VASP -GROMACS	-PBE GGA (DFT-D3) -GROMOS96	-Geometry optimization -MD	[230,232]	[192]
21	-GROMACS -AutoDock Tools -AutoDock Vina, Hex8.0.0 GROMACS	-AutoDockZN -AutoDock Vina, GROMOS96	-Energy minimization -Molecular docking -MD	[221,222,223,231]	[199]
22	-NAMD -Spartan04	-Charm CMAP FF -HF 3-21G	-MD -QM	[233,234]	[125]
23	-Gaussian 03 -AutoDock -AMBBER	-B3LYP/6311**G -AutoDockZN -PM3/Amberff14SB FF	-Geometry optimization -Molecular docking -QM/MM	[220,221,222,223,226,227,228]	[114]
24	-GROMACS -Gaussian 09	-CHARMM (MM) -GGA-D2 (QM)	-Geometry optimization -DFT calculations	[210,211,220,221,222,223,229]	[115]
25	-Maestro Schrödinger -AMBER Tools	-OPLS 2005 Glide -B3LYP/Amberff14SB	-Protein, ligand preparation (geometry optimization), molecular docking -QM/MM	[217,218,226,227,228]	[124]
26	-Crystal Predictor -Crystal Optimizer (Gaussian) -DMACRYS	-PBE0/631G(d,p)	-Conformations -Geometry optimization **CSP** -Intermolecular lattice energies	[220,235,236,237]	[150]
27	-GULP, DFTB+ -VASP	-optB88 level	-Geometry pre-optimization **CSP** -Geometry re-optimization	[232,235,236,237]	[181]
28	DMol3	DNP basis set, PBE GGA	Geometry, energy optimization	[238,239]	[182]
29	CASTEP	GGA PBE	DFT, NMR	[239,240]	[157]
30	CASTEP	GGA PBE	DFT, structure parameters calculation	[239,240]	[195]
31	Gaussian09W	B3LYP/631G(d)	DFT, IR	[238,241]	[164]
32	Gaussian09	M05-2X/6-311++G**	DFT, IR	[238,241]	[165]
33	Gaussian09W	B3LYP/6-31G (d,p)	DFT, Raman	[238,241]	[166]
34	Gaussian	B3LYP/6-31G(d,p)	DFT, IR	[238,241]	[167]

The most widely used are commercial codes: Maestro Schrödinger [217], CASTEP [240], GOLD [208], Gaussian [220], AMBER [226], SybylX, VASP [220]; academic codes: AutoDock [209], CHARMM [210], GROMACS [232]. (AMBER and CHARMM are names of both the codes and the force fields.) AMBER (Assisted Model Building with Energy Refinement), CHARMM (Chemistry at HARvard Macromolecular Mechanics), CSP (Crystal Structure Prediction), GAFF (General AMBER Force Field), Glide (Grid-based Ligand Docking with Energetics), GOLD (Genetic Optimization for Ligand Docking), GROMACS (GROningen MAchine for Chemical Simulations), HADDOCK (High Ambiguity Driven protein-protein DOCKing), HTVS (high throughput virtual screening), MM/GBSA (Molecular Mechanics/Generalized Born Surface Area), PCM (polarizable continuum model), VASP (Vienna ab initio Simulation Package), Glide SP (Standard Precision), XP (Extra Precision). References in the last column refer to articles already cited in this review. These are examples of application of the listed methods in (xeno)estrogens research. References in the fourth column refer to articles that describe the theoretical basis of the listed software and calculation methods.

**Table 4:** Comparison of the calculation methods used in (xeno)estrogen investigations.

Calculation Method	Pros and Capabilities	Cons and Limitations
Molecular docking	-Explanation of a molecular basis for protein–ligand binding -Relatively short calculation time -Enables virtual screening for active compounds	-Lower accuracy when compared to QM methods -Significant increase in time and complexity of calculations when combined with QM (QM/MM)
QSAR	-Evaluation of estrogenicity -No protein preparation needed	-No receptor–ligand binding data -Large set of high-quality experimental data needed to obtain accurate results
QM (DFT-D)	-High accuracy of calculations -Simulation of IR, Raman, NMR spectra -Thermodynamic calculations	-Long calculation time -A lot of computational power needed -Usually limited to small molecules and systems such as estrogen complexes, salts, co-crystals, etc.
QM/MM	-High accuracy of calculations in the binding area (QM) -Consideration of a whole complex (protein–ligand) with emphasis on the binding pocket	-Calculation time elongated due to QM -Limitation of the QM-calculated area
MD	-Simulation of dynamical processes -Possibility to perform DFT-MD	-Significantly longer time required

## Supplementary Materials

The following are available online at https://www.mdpi.com/1422-0067/21/17/6411/s1.

## Abbreviations

17β-HSD	17β-hydroxysteroid dehydrogenase
ADMET	Adsorption distribution metabolism elimination toxicity
BPA	Bisphenol A
CoMFA	Comparative molecular field analysis
DBD	DNA binding domain
DFT	Density functional theory
E1	Estrone
E2	Estradiol
E3	Estriol
E4	Estretrol
ER	Estrogen receptor
EDCs	Endocrine-disrupting chemicals
FEP	Free energy perturbation
H-K	Hohenberg–Kohn theorems
K-S	Kohn–Sham theorems
LBD	Ligand-binding domain
LIE	Linear interaction energy
MD	Molecular dynamics
MM	Molecular mechanics
PDB	Protein Data Bank
QM	Quantum mechanics
QSAR	Quantitative structure–activity relationship
RBA	Relative binding affinity
SERMs	Selective estrogen receptor modulators
SHBG	Sex hormone-binding globulin
STS	Sulfatase
SULT	Sulfotransferase
