# Supplementary material for: Application of Various Molecular Modelling Methods in the Study of Estrogens and Xenoestrogens
Source: Int J Mol Sci. 2020 Sep 3;21(17):6411. doi: 10.3390/ijms21176411 (PMC7504198; doi:10.3390/ijms21176411)
Supplement: Supplementary file 1 [file ijms-21-06411-s001.pdf]

International Journal of *Molecular Sciences*

Supplementary Materials

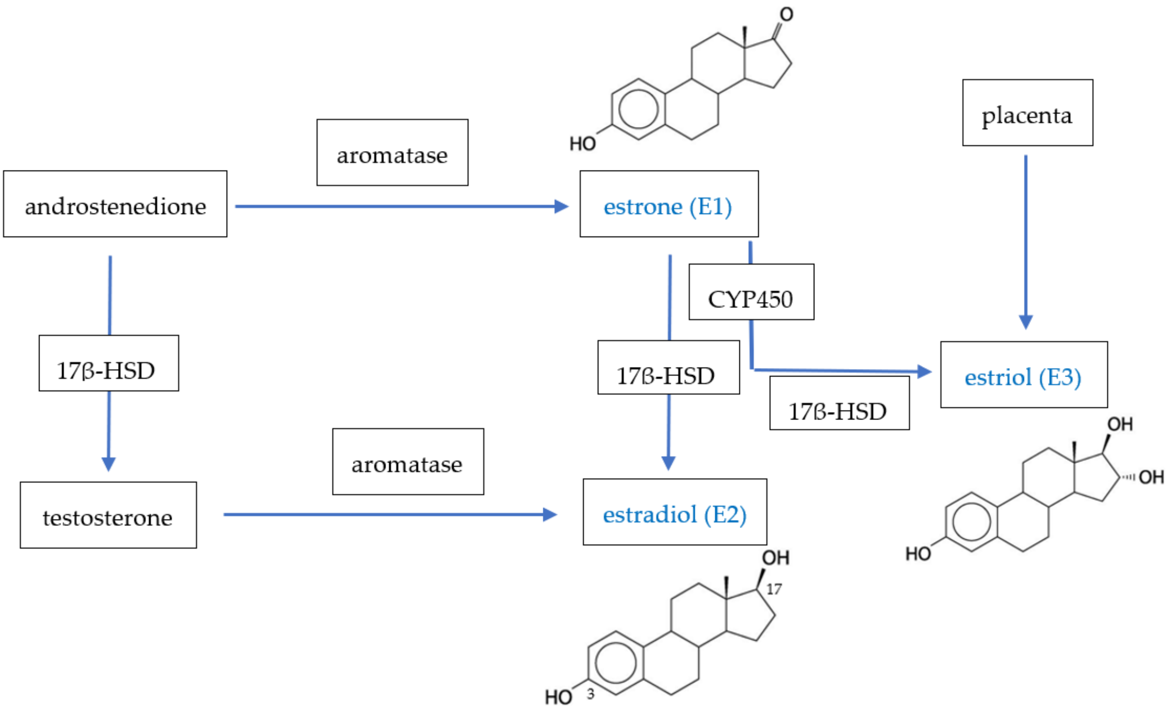

Figure S1. Mutual metabolic dependence between E1, E2, E3 and their structures. In E2 structure, carbon C3 and C17 have been marked. Based on:
Tofovic S.P..; Jackson E.K. Estradiol Metabolism: Crossroads in Pulmonary Arterial Hypertension. Int J Mol Sci. 2019, 21(1). pii: E116. doi: 10.3390/ijms21010116.

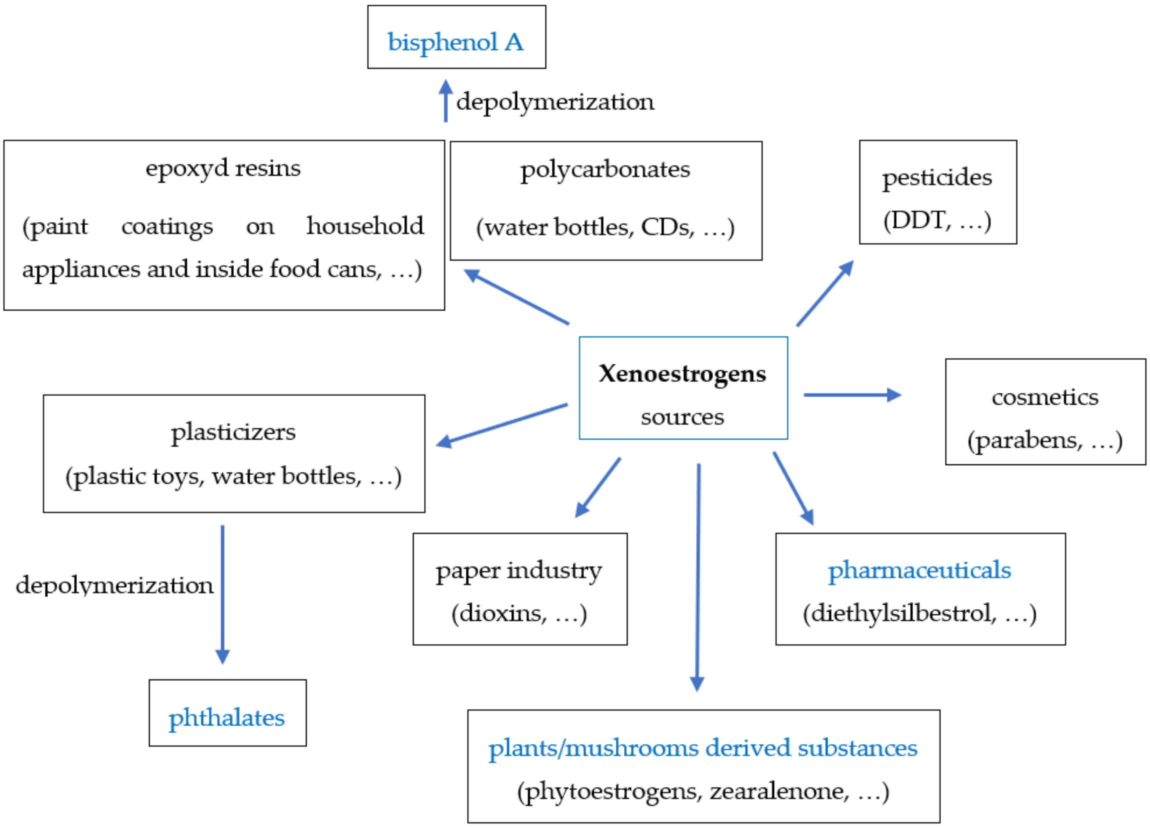

Figure S2. Main sources and types of xenoestrogens. In blue have been marked the ones frequently investigated with the application of *in silico* methods and, consequently, the ones mentioned in the main part of this review. Based on:

1. Cotterill, J.V.; Palazzolo, L.; Ridgway, C.; Price, N.; Rorije, E.; Moretto, A.; Peijenenberg, A.; Eberini, I. Predicting estrogen receptor binding of chemicals using a suite of *in silico* methods – Complementary approaches of (Q)SAR, molecular docking and molecular dynamics. *Toxicology and Applied Pharmacology* **2019**, *114630.* doi:10.1016/j.taap.2019.114630.
2. Kerdivel, G.; Habauzit, D.; Pakdel, F. Assessment and Molecular Actions of Endocrine-Disrupting Chemicals That Interfere with Estrogen Receptor Pathways. *International Journal of Endocrinology* **2013**, *1–14.* doi:10.1155/2013/501851.
